# Exploring Aggressive Behaviors in Greek Secondary Schools: Prevalence, Sociodemographic Factors, and Comparative Analysis with Elementary School Students

**DOI:** 10.3390/bs14050405

**Published:** 2024-05-13

**Authors:** Argyro Bourou, Petros Karkalousos, Anastasios G. Kriebardis, Effie Papageorgiou

**Affiliations:** Department of Biomedical Sciences, University of West Attica, 12243 Athens, Greece

**Keywords:** aggression, school bullying, secondary schools, Greek schools, school violence, teachers’ perspective

## Abstract

The main objectives of this study are to determine the prevalence of bullying in Greek secondary schools and detect the possible characteristics of bullies’ profiles in Greek school settings. A structured questionnaire was given to one hundred ninety-two (n = 192) educators at Greek junior high schools in urban and rural areas. The educators were asked to report the frequencies and forms of aggressive behavior observed during the 2022–2023 school year, the bullies’ sociodemographic characteristics, and ways of dealing with bullying episodes. The data are presented, after conducting statistical analyses, in comparison with data for elementary school students. The results revealed that higher rates of bullying were reported compared with elementary school children. Moreover, according to teachers’ observations, aggressive behavior is independent of a pupil’s diagnosis, but specific types are correlated significantly with a pupil’s gender, nationality, low academic performance, and popularity. Factor analysis showed two main factors of aggression types, where common points and differences with elementary school students are mentioned. Implementations for the prevention of school bullying are discussed.

## 1. Introduction

Bullying is a major problem in school communities worldwide [1]. School bullying is defined as a form of aggressive behavior on the part of the perpetrator that is characterized by repetition and an imbalance of power between the perpetrator and the victim and takes place in the school environment [2]. The negative acts towards the victim are expressed through various traditional forms such as physical or verbal attacks and relational/indirect bullying [3]. In addition to the traditional forms of aggression, cyberbullying has also occurred in the last decades [4,5], where the perpetrator retains his/her anonymity, and the offensive act can easily get publicity [6].

### 1.1. Individual Characteristics of Bully’s Personality

According to the literature, various personality traits contribute to aggressive behaviors in children. Many studies highlight the importance of gender in the manifestation of aggressive behavior. Adolescent boys are more likely to be perpetrators of direct bullying (physical or verbal), whereas adolescent girls tend to act indirectly [7,8,9]. Age also plays an important role in the occurrence of bullying incidents. As children get older, they are more likely to be involved in bullying episodes [9] or change the way they express their aggressive behavior [7]. More specifically, direct aggressive acts tend to be replaced by indirect ways of bullying and cyberbullying, depending on the age. Finally, aggression is more prevalent in secondary schools in comparison with primary schools, where hyperactivity is more prevalent [10].

Other personality traits like aggressiveness [11], hyperactivity and impulsivity [12], or social problem-solving skills and poor academic performance are linked to bullying behaviors [13]. Adolescent bullies also experience more anxiety and depression in comparison with their peers who are not involved with bullying [14]. Especially in adolescence, bullies are likely to dominate their peers or even be popular in their school setting [15].

Moreover, a low family socioeconomic status is a risk factor for being a bully [16]. Additionally, exposure to family harshness and violence [17] or poor parental supervision [15] contribute negatively to the manifestation of school bullying.

As far as ethnicity is concerned, the literature indicates that ethnical minorities are more likely to be bullies [1].

### 1.2. Prevalence of Bullying in Greek School Settings

Across the past twenty years, there has been a considerable body of research conducted in Greek schools focusing on the issue of bullying. The corresponding prevalence studies aimed to assess the frequency and the extent to which students were involved in bullying issues within a particular population or region. However, it is difficult to compare or group the results, since in each study, a different measurement tool, which is addressed to students, parents, or bystanders, is used.

The range of the prevalence of bullying varies approximately from 9.0% and 54% worldwide, and this large range is observed due to methodological issues [18]. According to Biswas [19], who conducted a study across the six World Health Organization regions, the pooled prevalence of victimization among adolescents was 30.5%, with the highest rate observed in the Eastern Mediterranean region (45.1%) and the lowest in Europe (8.4%).

Studies on bullying in Greek school settings also display a wide range of prevalence rates. To specify, in a cross-national study encompassing 40 countries [20], the findings revealed that 41.3% of Greek boys involved in the study were engaged in bullying incidents—either as bullies, victims, or both—occurring two or three times per month. Similarly, the rate among girls in Greece was notably high, with 28.3% participating in bullying episodes two or three times monthly, either as bullies, victims, or both. Lower percentages were reported in Sapouna’s study [21], where out of the sample pool comprising 1758 students from elementary and secondary schools, 8.2% were identified as victims, 5.8% as bullies, and 1.1% as both bullies and victims. Moreover, Magklara et al. [22] highlighted the importance of bullying among Greek adolescents, since, according to their findings, 26.4% of the adolescents studied were involved in a bullying episode at least once per month either as bullies, victims, or both. Another Greek study [23] highlighted the variations in the occurrence frequencies depending on who reported the incidents. Specifically, bystanders reported verbal aggression in 52.6% of cases, while bullies themselves reported 7.8%, and victims reported 6% of incidents involving verbal aggression.

Other studies conducted among Greek students estimated the rate of victimization in secondary education to be between 10% and 11% [18,24].

Despite differing methodologies and findings among studies, the majority of them concur that school bullying is a pervasive issue among pupils, and further studies are needed to gain a more comprehensive understanding of the phenomenon.

### 1.3. The Aim of the Present Study

The primary objectives of this study are to determine the prevalence of bullying within Greek junior high schools and reveal possible trends and risk factors associated with the episodes of bullying, as reported by teachers. As this study is an extension of the writers’ preceding research focused on elementary schools, we also aim to highlight the similarities and differences observed in pupils as they get older and how the teachers cope with aggression problems at primary and secondary education levels. Additionally, it is the first time that a study reports the frequency of bullying episodes from teachers’ perspectives on a national scale; this is important because teachers are usually the first observers of bullying behaviors and are considered a person of trust for students. In addition, teachers are more likely to witness students being bullied in educational settings and are also involved in handling bullying episodes [25].

To our knowledge, few past studies in the international literature have been conducted that approached bullying issues from teachers’ perspectives [26,27], and they mainly focused on the impact of the violent episodes witnessed on their pedagogical methods. Other studies report teachers’ responses regarding the bullying episodes they handle [28,29,30] with the use of hypothetical scenarios. The lack of studies where teachers are used as observers of bullying behaviors underlines the importance of our study. Additionally, there are few measurement tools designed for teachers, which complicates the observation process and enhances the development of such a tool.

Crothers and Levinson [24] note the existence of various assessment instruments for school bullying documented in the literature. These tools target either peers, such as the Peer Beliefs Inventory or Peer Relations Questionnaire (PRQ), or focus on bullies/victims themselves, like the Self-Rating Questionnaire on Aggressive Behavior (SQAB) or Olweus’s Bully/Victim Questionnaire (OBVQ). Our confidence lies in the effectiveness of our measurement tool, which is derived from the teacher rating instrument introduced by Dodge and Coie [25]. This adapted tool, tailored for the Greek school environment, is considered suitable for identifying bullying behaviors and delineating the characteristics of bullies, as reported by Greek teachers. Furthermore, we believe it holds promise as a prospective early-detection-of-violent-behavior tool for educators.

## 2. Materials and Methods

### 2.1. Participants

One hundred ninety-two teachers at Greek junior high schools (*n* = 192) replied to our questionnaire. The educators were requested to identify the frequency of the bullying episodes and the traits of aggressive adolescents (12–15 years old) they witnessed during the 2022–2023 academic year, along with the measures they employed. The data collection period was the last semester of the 2022–2023 academic year. The questionnaire was delivered to approximately over 1000 high schools; educators from one hundred ninety-two high schools responded voluntarily.

### 2.2. Measures

The questionnaire provided to the educators consisted of three sections. We should note that this questionnaire was also used for a previous study [31] that addressed elementary school teachers. The questionnaire was modified, with minor adjustments to suit the requirements of the current research focused on secondary education. To specify, the first section mainly included demographic information about the class (school region, number of boys or girls in the class, number of immigrants or diagnosed pupils, etc.).

The second section was answered only by teachers, who reported that they had witnessed a pupil who repeatedly acted aggressively. It concerned personal information about the aggressive pupil (age, gender, diagnosis if one existed, origin, family context, academic achievement, use of medication, popularity) as well as 12 statements that described the aggressive behaviors (physical/verbal aggression, direct/indirect aggression, etc.). The teachers were asked to report the frequency of the observed behaviors on a five-point scale ranging from 0 to 5 (never, rarely, sometimes, often, and very often). In particular, they answered questions such as “How often does the pupil threaten and bully his peers?” or “How often does the pupil blame his peers in fights?” These questions were based on the teacher rating instrument that was presented by Dodge and Coie [32], and their internal consistency was measured using a Cronbach’s alpha value of 0.758 [31].

Within the third section, the educators responded to questions about their approach to handling bullying incidents using a five-point scale. Specifically, the teachers were asked to assess the frequency with which they utilized various intervention methods in response to bullying episodes. These methods included actions like giving remarks or punishment, engaging in classroom discussions regarding the situation, providing reports to parents, or asking for advice from a school psychologist.

### 2.3. Procedure

Secondary school teachers were invited to answer a structured, non-standardized questionnaire. This questionnaire was distributed via email to approximately 1000 high schools in 28 prefectures, which were randomly selected in urban and rural areas across Greece. Prior to participation, the participants were briefed about the study’s objectives and provided their consent. All teachers completed the questionnaire voluntarily. No personal data about the participants or their pupils were requested. The questionnaire was firstly approved by the Research Ethics Committee of the University of West Attica.

### 2.4. Statistical Analysis

The statistical analyses of the data were conducted using Statistical Package for Social Sciences 27 (IBM SPSS 27), licensed under the University of West Attica. Descriptive statistics were used to collect aggressive children’s sociodemographic and personal characteristics. These characteristics were presented through means (M), medians, and standard deviations (SD). Additionally, we conducted chi-square tests to explore correlations among our categorical variables. The significance level (*p*-value) was set to 0.05.

All results are presented in comparison with the results of our previous study [31] regarding elementary school teachers.

## 3. Results

### 3.1. Descriptive Statistics

Our research was conducted among one hundred ninety-two educators of secondary education. Of these, one hundred sixty-two educators indicated that they have encountered aggressive children in their classroom, which accounts for 82.7% of the total number of respondents. In primary education [31], 206 out of 292 respondents reported having aggressive students, representing a percentage of 70.5%. It is important to note the growing trend of violent behavior among adolescents.

Table 1 presents the frequencies and percentages of aggressive children in regard to their age, gender, school region, and other social and demographic characteristics. We can observe that boys tend to exhibit more violent behaviors according to educators at both educational levels. Both percentages are extremely high: 90.1% for adolescents and 86.4% for younger pupils in comparison with girls. Moreover, although the percentage of aggressive pupils in urban regions is higher for primary education, in the case of secondary education, aggressive pupils are more evenly distributed between rural and urban regions. Significantly, a considerable portion of aggressive students exhibit lower academic performance across both educational levels, with a notably higher percentage observed (83.3%) within secondary education. Finally, in comparison with pupils of elementary education, the percentages are higher in aggressive pupils from a single-parent family (34.6%), with a foreign nationality (24.7%), and popularity among their peers (48.8%).

The means, medians, and standard deviations for each treatment that teachers use when they deal with bullying episodes are shown in Table 2. Giving parents a briefing about the incident is the most used technique for educators at the secondary level, while a remark given by the teacher is the less used technique. It is remarkable that in secondary education, punishments given by the principal are more frequently used in comparison with elementary education.

The means, medians, and standard deviations of the occurrence of bullying episodes are displayed in Table 3. It is noticeable that teachers of both educational levels report that aggressive episodes occur more frequently during break time. In addition, violent episodes happen with a greater frequency in secondary education classrooms in comparison with primary education classrooms.

Table 4 presents the means, medians, and standard deviations of the frequency of the types of aggressive behaviors reported by educators. The most frequently observed behavior among adolescent pupils is disobedience to teachers’ recommendations. We should also note that in comparison with primary education, while physical bullying occurs less frequently in secondary education, there is a significant occurrence of verbal bullying (direct and indirect) and cyberbullying. Difficulty in anger management and the expression of anger are also highly observed by educators.

**Figure 1:**
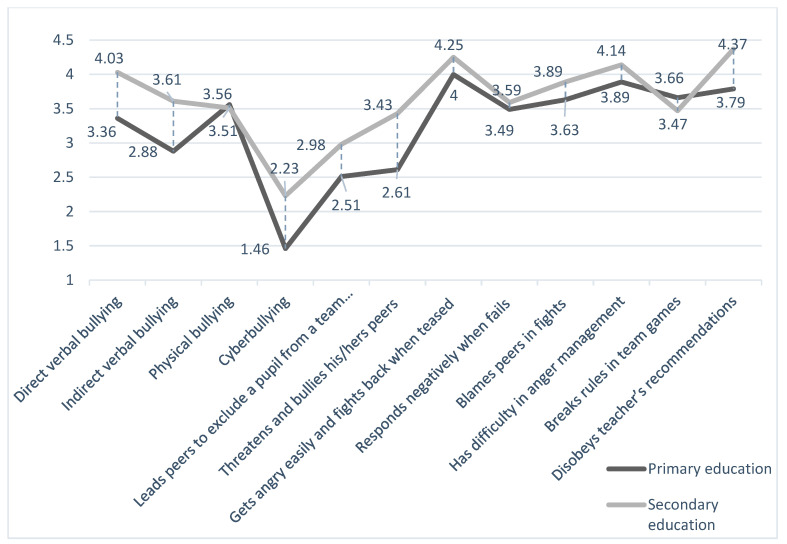
Means of frequencies of different forms of aggression in primary and secondary education.

In Figure 1, the means of the frequencies of different forms of aggression in primary and secondary education are depicted. All types of aggressive behaviors tend to occur more frequently during secondary education, except for breaking rules in team games and physical bullying, where a minor decrease is noted.

### 3.2. Correlations

Chi-square tests were conducted between all forms of aggressive behaviors and children’s sociodemographic characteristics in order to explore significant correlations (a < 0.05); the results are displayed in Table 5. The characteristics taken into consideration were the gender, age, nationality, diagnosis, family context, academic performance, use of medication, and popularity. Gender is only significantly correlated with difficulty in anger management, which is a common point with primary education, but it is not correlated with physical violence. Physical bullying is not significantly associated with any other sociodemographic factors. A pupil’s nationality is correlated with threatening and bullying his/her peers. Children who exhibit indirect verbal bullying tend to have low academic performance and be taking medication. Aggressive children with popularity among their peers are likely to lead their peers to exclude a pupil from a team activity. Aggressive children from a single-parent family are likely to respond negatively when they fail.

A pupil’s diagnosis is not significantly correlated with any form of aggressive behavior, and this is a main difference that is noted between primary and secondary education.

### 3.3. Factor Analysis

Factor analysis was conducted in order to identify the primary variables that contribute to aggressive behaviors among elementary school pupils. The questionnaire used comprised 12 variables, each describing a unique form of aggressive behavior. The variables are clearly shown in Table 6. Additionally, communality values that assess how well variables are explained by the factors are also depicted in Table 6.

In this study, the KMO (Kaiser–Meyer–Olkin Measure of Sampling Adequacy) was 0.878, which indicated that the sample was adequate for performing factor analysis, and the Bartlett’s Test of Sphericity result was significant (*p* = 0.000).

Table 7 shows factor loadings after using Varimax with the Kaiser Normalization rotation method, with a significant factor criterion of 0.6.

As shown in Table 7, factor 1 is composed of items 7, 8, 10, and 12. It can be labeled “anger and negative response”, and it is mainly loaded by difficulty in anger management (10), disobedience to teachers’ recommendations (12), negative response when fails (7) and becoming angry easily and fighting back when teased (8).

Factor 2 consists of items 2 (indirect verbal bullying), 4 (cyberbullying), 5 (leading peers to exclude a pupil from a team activity), and 6 (threatening and bullying). This factor can be labeled “indirect active bullying”.

## 4. Discussion

The current study focused on aggressive behaviors observed by educators of secondary education in Greek schools. The results presented are displayed in comparison with those from the elementary education. The objective of this research was to offer a clearer understanding of the nature of bullying incidents observed by educators of Greek adolescents from the teachers’ perspectives, while simultaneously identifying any variations or commonalities between aggressive episodes that occur in primary and secondary education in Greek school settings.

Bourou and Papageorgiou, in their previous study [31], examined the prevalence of bullying in Greek school settings from the point of view of teachers at elementary schools. Specifically, they outlined the aggressor’s characteristics and found that boys and pupils with low academic performance tended to act aggressively more frequently, while aggressive behaviors were not associated with the aggressor’s age, nationality, or family status. Moreover, after conducting factor analysis, they revealed four dominant factors that could describe the aggressive behaviors of elementary school pupils: “offensive behavior”, “misconduct behavior”, “outbursts of anger”, and “cyber-harassing behavior”.

The main contribution of the present study is the mapping of violent episodes in secondary education in Greek school settings, as perceived by teachers, and the detection of the tendency that exists between certain risk factors (i.e., bully’s sociodemographic characteristics) and aggressive behaviors. Additionally, the results are presented in comparison with prior results for elementary education. It is important to note that no other national study has been conducted using a questionnaire completed by teachers, who are believed to have direct contact with students and are better equipped to respond to different bullying situations [25].

In Table 1, it is evident that in both primary and secondary education, a higher percentage of boys (86.4% and 90.1%, respectively) are involved in bullying episodes, as indicated by the teachers’ responses. These findings are consistent with the prevalence of boys as bullies reported in other studies conducted globally [20,33].

Overall, higher rates of bullying are reported by teachers in the secondary education sector compared to the primary education sector, as shown in Figure 1. According to Fujikawa et al. [34], bullying across early adolescence is more frequent for both genders and particularly more persistent in girls. Other studies also underline that all types of bullying may peak in middle school [35,36].

A significant increase in the frequency of cyberbullying is being noticed by teachers at secondary schools in comparison with primary schools. This finding may be explained because adolescents have greater access to technological means, although according to the existing literature, the association between age and cyberbullying is not consistent across studies [37]. According to Basile et al. [38], electronic bullying is one of the main forms of violence expressed among adolescents in the U.S., since 15.7% of the study participants reported that they had been cyber-bullied during the previous twelve months. Additionally, according to Flannery [39], although the prevalence of traditional bullying has remained constant, the occurrence of cyberbullying has escalated significantly the last decade.

Moreover, according to our results, verbal bullying, direct and indirect, tends to dominate among Greek adolescents, and this is consistent with previous studies in Greece that reported similar results [18]. In other countries, the prevalence of verbal bullying is also notably high in adolescence, as is referred to in the study of Bachler et al. [40], where 53.6% of the study participants reported being verbally bullied, and in the study of Maharjan et al. [41], where the rates of verbal bullying were 75.8% and of relational bullying, 57.7%. Additionally, physical bullying tends to be replaced by verbal bullying in higher grades [42] or decline in adolescence, because indirect forms of bullying like anger and hostility occur more frequently [43]. Threatening and bullying peers also occurs at a greater frequency in secondary education according to our findings. This is consistent with the general increase in verbal bullying, which could also include direct threats among peers.

Both in the primary and secondary sector, difficulty in anger management and anger expression are reported as frequently noticeable among pupils. Anger contributes positively to physical and verbal aggression [44] and is connected to social maladjustments and anti-social behaviors in adolescence [45].

As far as the aggressor’s sociodemographic characteristics are concerned, as is shown in Table 5, gender is only associated strongly with anger management and not with physical violence as in primary education. This finding is in contrast with previous studies, where boys tend to be more involved in physical bullying and girls in relational bullying [8,36]. According to our results, low academic performance is strongly associated with indirect verbal bullying while in primary education it was associated with physical bullying (Table 5). In previous studies, low academic performance also seems to be a risk factor for being a bully or a bully victim [46,47]. Physical bullying may not be correlated with gender and a low academic profile, because it tends to maintain a consistent level of frequency, according to our results, in regard to other forms of aggression. Thus, Greek adolescents tend to express their aggression with other forms of bullying and not physical power.

An aggressor’s nationality is correlated to threatening peers. This is consistent with other studies, where it is mentioned that bullies may have an immigrant background [48,49,50]. This finding does not agree with the corresponding finding in elementary education and could be explained because adolescents may express their national identity more intensively in comparison with primary education students.

Our results indicate that an aggressor’s popularity is strongly correlated with leading peers to the social exclusion of a victim. The literature also supports that a bully’s popularity is a risk factor for school violence [51].

Our research demonstrates that the diagnosis of a developmental or learning disorder among bullies is separate from exhibiting aggressive behavior. This result can be attributed to the high prevalence of learning disabilities and autistic disorders in Greek school settings, while the previous literature primarily suggests a correlation between hyperactivity and impulsivity and perpetrator’s aggressive behavior [1].

The current study reveals that Greek educators, both in primary and secondary education, assert that bullying incidents frequently happen in the schoolyard during breaks rather than in class (Table 3). This outcome aligns with previous research, as children have more chances to engage in or initiate aggressive behaviors during breaks [52]. Furthermore, pupils feel less insecurity and more vulnerability to aggressive behaviors during recess periods, where supervision is less rigorous than in the classroom setting [53].

Our study highlights that teachers of secondary education are more likely to use more punitive measures (punishment given by the educator or the principal) in comparison with primary education to deal with violent episodes in Greek school settings, as shown in Table 2. This could be explained because of the higher prevalence of violent episodes in middle school, the absence of a national anti-bullying intervention program, or the lack of educators’ training on handling pupils’ aggression. This finding agrees with previous findings, where it is mentioned that the majority of teachers prefer authority-based interventions and not alternative approaches to bullying episodes [28]. Additionally, in order to effectively deal with issues of violence in the classroom, the teachers’ participation in training programs is considered crucial [54]. However, in some cases, Greek teachers also try to enhance the school climate using the advice of a specialist.

Through the factor analysis conducted (Table 7), we attempted to map the tendency of the violent behaviors exhibited by adolescent pupils in Greek school settings. To specify, two main factors were revealed for the adolescent students instead of four factors that were reported for elementary schools. The first factor, named “anger and negative response” describes behaviors such as anger outbursts with or without a prompt, disobedience to adults, and negative response to peers. This factor presents common features with the factor “outbursts of anger” in elementary schools. This finding demonstrates that difficulties in anger management seem to be a current issue in Greek schools. This is consistent with other studies globally, where it is highlighted that the lack of anger control is an upcoming issue during adolescence and can provoke violent behaviors [55]. The second factor, labeled “indirect active bullying” comprises actions of bullying with the intention to harm peers by various means such as cyberbullying, indirect verbal bullying, or peer exclusion from a team activity. In comparison with the findings of elementary schools, this factor could represent a combination of “offensive behavior” and “cyber—harassing behavior”.

The absence of direct verbal bullying and physical bullying in the factors described could be explained because as pupils get older, they express their aggression with more indirect forms [7].

### Implementations and Limitations

Educators need to be aware of the types of violent behaviors that pupils may display. The more informed they are, the more effectively they can handle bullying issues. Moreover, it is crucial for educators to be able to distinguish the form of violence used each time, so that they can apply specialized measures that are appropriate for each case. For instance, adolescents may manage their anger if they are taught problem-solving, communication, and social skills [56,57]. Additionally, it is important that educators detect and handle issues of anger management early in order to prevent negative behaviors [55]. Furthermore, parents’ restrictive measures for the use of electronic devices or the students’ awareness about internet risks may prevent cyberbullying [58]. In light of the findings from our current study, it is possible to develop an assessment tool that would enable teachers to effectively observe and report bullying behaviors and quickly identify the specific type of bullying of each student. Such a tool would facilitate the implementation of appropriate intervention measures.

Additionally, a crucial aspect to consider, according to our results, is that the proportion of questionnaires delivered and not completed by educators may imply a lack of awareness and difficulty on the part of teachers in identifying aggressive behaviors. It is imperative that this aspect is taken into account and results in the development of educator training programs. This aspect also underlines the necessity of an assessment tool for teachers.

Regarding the limitations of the current study, the results should be interpreted cautiously due to insufficient awareness among teachers regarding the various forms of bullying in school-aged children. Some educators may misinterpret a violent incident and react passively, or they may have difficulty identifying aggressive incidents and adequately comprehending the severity of the problem in school settings regarding their age, education level, gender, or other personal factors. Additionally, the study relied on self-reported measures and utilized a convenient sample.

## 5. Conclusions

The main purposes of this study were to examine the extent of bullying in Greek secondary schools and detect differences and common points between primary and secondary education from teachers’ perspectives. It is the authors’ belief that the results deduced from the current comparison have been reported for the first time, since no other research has been conducted on a national scale using a teacher questionnaire. Specifically, the current research findings reveal that aggressive behaviors tend to escalate as students grow older. As a result, educators at the secondary school level are more likely to implement stricter measures to handle violent incidents. Moreover, adolescents have the tendency to exhibit their aggressive behavior in various forms, with expressions of anger and cyberbullying being the most prevalent. The prevalence of anger management issues and verbal bullying remains high in secondary education, while physical violence has decreased slightly. It is important for teachers to be aware of different types of bullies in order to easily recognize violent episodes in school settings.

It would be beneficial, for future research, to compare the aggression types described for Greek students with the outcomes of similar international studies. Finally, studies in a wider range that explore the extent to which educators are aware of bullying issues must be conducted in future research, with the goal of contributing to the development of effective national prevention programs that protect students’ mental health.

## Figures and Tables

**Table 1:** Sociodemographic characteristics of aggressive children in elementary and secondary schools.

	Primary Education ^a^		Secondary Education	
Baseline Characteristic	*n*	%	*n*	%
Gender				
Female	28	13.6	16	9.9
Male	178	86.4	146	90.1
Age				
5	53	25.7		
6–8	73	35.4		
9–11	79	38.3		
12–15			162	100
Region				
Urban	115	56.0	82	50.6
Rural	91	43.5	80	49.4
Single-parent family ^b^	42	20.3	56	34.6
Foreign parents ^b^	34	16.4	40	24.7
Diagnosed children ^b^	55	26.6	38	23.5
Popular among his/her peers ^b^	79	38.2	79	48.8
Low academic performance ^b^	110	53.1	135	83.3
Taking medication ^b^	16	7.7	11	6.8

Note: N = 207 (primary education) and N = 162 (secondary education); *n* stands for each condition. ^a^ Results concerning elementary education, presented in [31]. ^b^ Reflects the number and percentage of participants answering “yes” to this question.

**Table 2:** Means, medians, and standards deviations about teacher’s way of dealing with bullying episodes.

How Teachers Deal with Bullying Episodes	Mean		Median		Standard Deviation	
	PE	SE	PE	SE	PE	SE
Punishment given by the teacher	2.66	3.51	3.00	3.00	1.000	1.001
Punishment given by the principal	2.17	3.53	2.00	4.00	1.003	1.148
Discussion about bullying episode in the classroom	4.25	3.77	4.00	4.00	0.786	1.118
Give parents briefing about the incident	3.73	4.17	4.00	5.00	1.023	1.049
Search for a specialist’s advice (e.g., psychologist)	3.12	3.72	3.00	4.00	1.295	1.148

Note: (PE = primary education and SE = secondary education). Results concerning elementary education, presented in [31].

**Table 3:** Means, medians, and standards deviations for the place that bullying episodes occur more often according to teachers.

Occurrence of Bullying Episode	Mean		Median		Standard Deviation	
	PE	SE	PE	SE	PE	SE
During class	3.33	3.86	3.00	4.00	1.056	0.964
During break	4.01	3.98	4.00	4.00	0.789	0.952

Note: (PE = primary education and SE = secondary education). Results concerning elementary education, presented in [31].

**Table 4:** Prevalence of different types of aggressive behaviors.

Forms of Aggressive Behavior	Mean		Median		Standard Deviation	
	PE	SE	PE	SE	PE	SE
1. Direct verbal bullying	3.36	4.03	3.00	4.00	1.063	0.908
2. Indirect verbal bullying	2.88	3.61	3.00	4.00	1.181	1.116
3. Physical bullying	3.56	3.51	4.00	4.00	0.970	1.107
4. Cyberbullying	1.46	2.23	1.00	2.00	0.881	1.195
5. Leads peers to exclude a pupil from a team activity	2.51	2.98	3.00	3.00	1.132	1.068
6. Threatens and bullies his/her peers	2.61	3.43	3.00	3.00	1.115	1.039
7. Gets angry easily and fights back when teased	4.00	4.25	4.00	4.00	0.847	0.829
8. Responds negatively when fails	3.49	3.59	4.00	4.00	1.121	1.101
9. Blames peers in fights	3.63	3.89	4.00	4.00	1.137	0.985
10. Has difficulty in anger management	3.89	4.14	4.00	4.00	1.016	0.916
11. Breaks rules in team games	3.66	3.47	4.00	4.00	1.034	1.073
12. Disobeys teacher’s recommendations	3.79	4.37	4.00	5.00	1.010	0.826

Note: (PE = primary education and SE = secondary education). Results concerning elementary education, presented in [31].

**Table 5:** Associations (*p*-values) of aggressor’s sociodemographic characteristics and forms of aggressive behavior after conducting chi-square tests.

	Gender	Nationality	Diagnosis	Single-Parent Family	Low Academic Performance	Taking Medication	Popular among Peers
Direct verbal bullying	0.969	0.880	0.894	0.258	0.664	0.780	0.686
Indirect verbal bullying	0.769	0.414	0.540	0.946	**0.007** (0.409)	**0.001** (0.620)	0.628
Physical bullying	0.405 **(0.000)**	0.924	0.795	0.605	0.380 **(0.018)**	0.663	0.490
Cyberbullying	0.290	0.755	0.456	0.341	0.371	0.331	0.207
Leads peers to exclude a pupil from a team activity	0.241	0.557	0.151 **(0.025)**	0.391 **(0.043)**	0.927	0.436	**0.013 (0.041)**
Threatens and bullies his/her peers	0.674	**0.036** (0.759)	0.619	0.565	0.753	0.581	0.279
Gets angry easily and fights back when teased	0.864 **(0.000)**	0.420	0.291	0.144	0.965	0.337	0.385
Responds negatively when fails	0.269	0.912	0.422 **(<0.001)**	**0.014** (0.593)	0.755	0.882	0.502 **(0.017)**
Blames peers in fights	0.111	0.515	0.343 **(0.001)**	0.553	0.135	0.319	0.476
Has difficulty in anger management	**0.033 (0.007)**	0.472	0.240 **(<0.001)**	0.056	0.142	0.516 **(0.015)**	0.454 **(0.001)**
Breaks rules in team games	0.540	0.948 **(0.030)**	0.676 **(<0.001)**	**0.047** (0.739)	0.166	0.320	0.449 **(0.001)**
Disobeys teacher’s recommendations	0.755	0.523	0.412	0.657	0.594	0.517	0.126 **(0.041)**

Note: *p*-values < 0.05 are noted in bold. Associations (*p*-values) of aggressor’s sociodemographic characteristics and forms of aggressive behavior in primary education are noted in brackets [31].

**Table 6:** Communality values.

	Initial	Extraction
1. Direct verbal bullying	1.000	0.619
2. Indirect verbal bullying	1.000	0.574
3. Physical bullying	1.000	0.563
4. Cyberbullying	1.000	0.678
5. Leads peers to exclude a pupil from a team activity	1.000	0.612
6. Threatens and bullies	1.000	0.629
7. Responds negatively when fails	1.000	0.635
8. Becomes angry easily and fights back when teased	1.000	0.527
9. Blames peers in fights	1.000	0.480
10. Difficulty in anger management	1.000	0.699
11. Breaks rules in team games	1.000	0.345
12. Disobeys teachers’ recommendations	1.000	0.612

Note: Extraction method: Principal Component Analysis.

**Table 7:** Factor loadings.

	Component	
	1	2
1. Direct verbal bullying	0.525	0.586
2. Indirect verbal bullying		**0.666**
3. Physical bullying	0.579	0.478
4. Cyberbullying		**0.823**
5. Leads peers to exclude a pupil from a team activity		**0.779**
6. Threatens and bullies	0.435	**0.663**
7. Responds negatively when fails	**0.754**	
8. Becomes angry easily and fights back when teased	**0.632**	
9. Blames peers in fights	0.566	0.400
10. Difficulty in anger management	**0.831**	
11. Breaks rules in team games		0.468
12. Disobeys teachers’ recommendations	**0.781**	

Note: Extraction method: Principal Component Analysis. Rotation method: Varimax with Kaiser Normalization. Rotation converged in 3 iterations. Significant factor criterion of 0.6 is noted in bold.

## Data Availability

The data that support the findings of this study are available from the corresponding author upon reasonable request.
